# Determinants of unintended pregnancy among women attending antenatal clinic at Kenyatta National Hospital.

**DOI:** 10.12688/f1000research.108815.1

**Published:** 2022-05-27

**Authors:** Rose Ojuok, Dr. Daniel Nyamongo, Dr. Joseph Mutai

**Affiliations:** 1 Environmental Health and Disease Control, Institute of Tropical Medicine and Infectious Diseases (ITROMID), Jomo Kenyatta University of Agriculture and Technology, Nairobi, Nairobi, 62000-00200, Kenya; 2 Environmental Health and Disease control, Jomo Kenyatta University of Agriculture and Technology, Nairobi, Nairobi, P.O BOX 62000-00200, Kenya; 3 Centre for Public Health Research (CPHR), Kenya Medical Research Institute, Nairobi, Nairobi, 54840-00200, Kenya

**Keywords:** Determinants, unintended pregnancy, family planning, 18-49 years pregnant women

## Abstract

**Background: ** Unintended pregnancy predisposes women of child-bearing age to risk factors like maternal deaths, poor child outcomes, mental illness because of stress, risky abortion, and vertical transmission of HIV. According to the Kenya Demographic Health Survey in 2014, 34% of the pregnancies were unintended and in the year 2020 it rose to 41.9% (Monitoring, 2020).  Determinants of unintended pregnancy among women attending antenatal clinics in Kenya is diverse and is poorly understood due to no representative information.

The objective of the study was to determine the factors associated with unintended pregnancy among women attending antenatal clinic particularly their individual factors, family planning practices and health facility-based factors.

**Method**:  A cross-sectional study design. Data was collected using a structured administered questionnaire from 227 participants. The proportion and determinants of unintended pregnancy was derived using bivariate analysis and multivariate logistic regressions.

**Results:** In this study, a third (29.9%) of the pregnant women reported that their existing gravidity was unintended. Individual factors such as age less than 25 years [AOR 8.1 (95% CI 1.4-48.6)), *p*=0.001], use of contraceptive method [AOR 7.9 (95% CI 2.5-25.0), *p*<0.001] and the woman being the sole decision-maker on when to get pregnant [AOR 3.8 (95% CI 1.3-11.2), *p*=0.014] were significantly associated with unintended pregnancy.

**Conclusion:** The study area had quite a significant proportion of unintended pregnancy underscoring the need for health facilities to enhance targeted contraceptive counselling during antenatal and postnatal clinics. Reinforcing effective utilization of family planning services in the pursuit to decrease unintended pregnancy not only in Nairobi but also in Kenya.

## Introduction

Worldwide, women upon reaching a childbearing age are at risk of experiencing a pregnancy that is unintended or intended. A pregnancy is unintended if it occurs when a child is not desired (unwanted) or not anticipated at that particular time (mistimed) ( Ameyaw *et al.*, 2019). Globally, 210 million pregnancies take place annually out of which about 75-80 million are unintended ( Ali, Tikmani, *et al.*, 2016). In Africa, out of 49.1 million pregnancies, unintended pregnancies were 39%. Sub-Saharan Africa record about 14 million unintended pregnancy annually ( Ameyaw *et al.*, 2019). Presently, developed and developing countries share in this problem of unintended pregnancy though the prevalence is greater in the former than the latter (Singh *et al.*, 2010). As from 1995-2008, unintended pregnancy reduced by 20% with developed countries having 29% decrease compared to developing countries that had 20%.Despite the reduction in these rates, the existence of unintended pregnancy was still comparatively elevated ( Ali & Ali, 2014).

Over the years, globally, there has remained a decline in the number of unintended pregnancy especially between 2010 and 2014 with its prevalence reducing from 30% to 16% ( Ameyaw *et al.*, 2019). In Kenya, the prevalence of unintended pregnancy was at 43% according to the KDHS 2008-2009 among married women ( Ikamari *et al.*, 2013) and at 34.4% in 2014 ( Ameyaw *et al.*, 2019). Unintended pregnancy is a public health problem that cuts across all the countries in the world due to its negative health and socio-economic outcomes to the mother, family, and society in general. It predisposes women of child-bearing age to risk factors like risky abortion, maternal mortality, poor child outcomes, mental illness as a result of stress and vertical transmission of HIV ( Ameyaw *et al.*, 2019). It affects adolescents or teenagers, married and unmarried women of reproductive age, and because of the humiliation or dishonor that sometimes it comes with, particularly teenagers or young women become obliged to undertake unsafe abortion. Out of the unintended pregnancy that occurs in Kenya, 14% of them end up in unsafe abortions claiming 2600 women and girls’ lives annually (Mumah *et al.*, 2014). The key influencing factor for risky abortion which has led to high maternal mortality and morbidity in Kenya is unintended pregnancy ( Obiero & Mwai, 2018). A report by Allan Guttmacher Institute (2012) indicates that unsafe abortion is prevalent in East Africa especially in Kenya where it is restricted and stigmatized. Unsafe abortion claims 500 women lives per 100,000 ( Hussain, 2012).

Unintended pregnancy has adverse maternal and neonatal outcomes which include death and disease related to induced and unsafe abortions, poor and delayed utilization of maternal health care service, poor child birth outcomes, low physical maternal health and poor maternal mental health ( Exavery *et al.*, 2014). Kenya seeks to guarantee that every gravidity is planned, every birth safe and each young person reaches their potential by the year 2030 guided by the Sustainable Development goals. This they will achieve by putting an end to unmet need for contraception and preventable maternal deaths hence will positively impact on inadvertent pregnancy ( UNFPA, 2015).

There are studies that have been done in the past to evaluate the prevalence, predictors and correlates of unintended pregnancy like in Sub-Saharan Africa ( Ameyaw *et al.*, 2019), narrative review of this problem in developing countries ( Ali, Tikmani, *et al.*, 2016), Ethiopia ( Hamdela *et al.*, 2012), Tanzania (Calvert *et al.*, 2013), Nigeria ( Sedgh *et al.*, 2006), Ghana ( Nyarko, 2019) and Kenya ( Ikamari *et al.*, 2013) ( Mumah *et al.*, 2013). With wide spread search on the work that has been done on this problem in Kenya, it was evident that women of childbearing age in slum and non-slum settlement had high prevalence of unintended pregnancy irrespective of their income status or wealth index of the household and educational accomplishment. Despite the accessibility of effective methods of contraceptives, incorrect and inconsistence use and contraceptive method failure also contribute to unintended pregnancy.

Determinants of unintended pregnancy among women of reproductive age attending antenatal clinics in Nairobi, Kenya is diverse in addition is poorly understood due to no representative information. Unintended pregnancy has extreme side effects for the mother and child and the best point for government, supporting public health partners and stakeholders to intervene is at this point. The proportion of women attending antenatal clinics in Kenya is said to be about 94%, this gives health care providers an opportunity to assess adequately case by case the type of pregnancies that these women have. This study sought to find out the level of unintended pregnancy among women attending antenatal care in Nairobi and the associating factors.

## Methods

### Study design and data source

A cross-sectional study design was conducted to assess the prevalence and determinants of unintended pregnancy among expectant women aged 18-49 years old attending antenatal clinic at Kenyatta National Hospital. It excluded any woman aged below 18 years, in labor or had complications, declined to consent and those who were unable to communicate (who could not hear, speak, or mentally challenged). Kenyatta National Hospital is the largest Level 6 referral hospital in Nairobi, Kenya and offers services to people from various parts of the country and its environs. According to the facility daily registry, about 1200 women attend the antenatal clinic every month at Kenyatta National Hospital antenatal clinic.

### Study population

All women attending the Kenyatta National Hospital antenatal clinic during the time of study were the study population.

### Sample size determination and sampling procedure

The Fisher *et al* 1998 formula n=(Z ^2^ p q) **÷**d ^2^ was applied with assumption of; 24% of the population proportion had prevalence of unintended pregnancy in Nairobi ( Ikamari *et al.*, 2013), 95% confidence interval, marginal error of 5% (0.05) to calculate the sample size. The target population was less than 10,000 hence used nf = n/1+(n/N). Sample size was 227 pregnant women.

Systematic sampling method was used to choose study participants from the antenatal clinic registration book that contains a list of all the pregnant women (sample frame) visiting the ANC clinic. The sampling fraction K was calculated by dividing the anticipated number of pregnant women who visited the ANC clinic monthly which was approximately 1200, by the calculated sample size (227). The study participants were selected at every ‘Kth’ intervals which was 5. So after every five pregnant women, one study participant was recruited for the study by the nurse after triaging till the sample size was obtained ( Daniel & Cross, 2013).

### Variables

To create an unintended pregnancy as the outcome variable, a dichotomous outcome was created by coding participant’s response on whether they wanted to become pregnant then (if yes, it was intended or wanted) (0) or wanted to become pregnant later (mistimed) or did not want children (unwanted) (1). The pregnancy was categorized into two groups: the intended and unintended (mistimed and unwanted). The predictor variables were the individual factors like age, marital status, educational level, work status, religion, occupation, and facility-based factors. Likert scale for rating was used to determine the facility-based factors associated with unintended pregnancy. The responses were rated ranging from one to six which stood for strongly disagree, disagree, slightly disagree, slightly agree, agree, and strongly agree, respectively. The first three represented fifty percent negative response and the latter three stood for fifty percent positive response. Practices on family planning was also determined in the study as the intervening variable to determine the use of contraceptive, types that they preferred and where they sought the family planning services. The response rate of the study was good and various variables like socio-demographics, family planning practices and respondents’ perspectives on health care services were examined at the same time.

### Ethical approval and consent to participate

Kenyatta National Hospital/ University of Nairobi Ethics and Research Committee (KNH/UON ERC) reviewed and permitted this study to be done at the Kenyatta National Hospital antenatal clinic. The KNH/UON ERC approval number was P421/05/2019. There were two nurses at the triage one of whom was a research assistant responsible for guiding the eligible participants into a private room for the consenting process.

Informed consent was obtained from the eligible study participants prior to enrolling them into the study. The written informed consent was translated into Kiswahili. During the consenting process, the written informed consent was explained in detail either in Kiswahili or English depending on the language that was comfortable to the participant. Anonymity and confidentiality were maintained throughout the data collection, management, and dissemination.

### Data analysis

In the design and execution of this study, great effort was set into undertaking a trial run, keeping the data collection protocol simple and easy to administer, communicating details of the study to the participants to lessen the occurrence of missing data. Quantitative data entry was done using a Microsoft Office excel 2010 thereafter coding and analysis based on Statistical Package for Social Sciences (SPSS) version 21. Descriptive and bivariate analysis was used primarily to check the proportion of unintended pregnancy among the predictor variables. Multivariate logistic regression was further used to determine the association between unintended pregnancy and sociodemographic factors, practices on family planning and health facility-based factors. Variables with substantial association were identified based on OR (odds ratio) with 95% confidence interval (CI) and those having association with the dependent variable with a p-value less than 0.05 were entered to Multivariate Logistic regression for controlling possible effects of confounders during analysis.

## Results

### Background and reproductive health characteristics

Table 1 presents selected individual factors, facility-based factors and practices of currently women attending antenatal clinic at Kenyatta National Hospital. Majority (78.4%) of the women were aged between 25 and 39 years old. Most of the women (86.8%) lived in the urban areas, 58.6% had post-secondary education and 83.3% were married or cohabiting while 16.7% were not living with a spouse. Partner’s level of education was mainly post-secondary in 63% and a half (50.7%) of the women were employees in the private sector. Of all the study participants, 80.7% were multiparous with 62.8% of them having had more than three births. 29.9% of the pregnancies were unintended out of which 83.8% were mistimed and 16.2% were unwanted pregnancy. 70% of the pregnancies the women had been intended.

**Table 1.** Sociodemographic characteristics of the study participants.

Categorical variable	Frequency (n=227)	Percentage (%)
**Age in years**		
18-24	33	14.5
25-29	57	25.1
30-34	80	35.2
35-39	41	18.1
40-44	14	6.2
45-49	2	0.9
**Residence**		
Urban	197	86.8
Rural	30	13.2
**Education level**		
Primary	17	7.5
Secondary	77	33.9
Post-secondary	133	58.6
**Marital status**		
Single	35	15.4
Married	184	81.1
Separated	3	1.3
Cohabiting	5	2.2
**Lives with spouse**		
Yes	189	83.3
No	38	16.7
**Religion**		
Catholic	104	45.8
Protestant	107	47.2
Muslim	8	3.5
Others	8	3.5
**Partner’s education level**		
None	3	1.3
Primary	8	3.5
Secondary	62	27.3
Post-secondary	143	63
Do not know	11	4.8
**Occupation**		
Student	11	4.8
Merchant	24	10.6
Housewife	54	23.8
Government employee	6	2.6
Private employee	115	50.7
Daily laborer	17	7.5
**Gravidity**		
Once	44	19.3
2 times	68	30
3 and more	115	50.7
**Parity**		
Zero	38	16.7
Once	63	27.8
2 times	72	31.7
3 and more	54	23.8
**Frequency of seeking FP services in health facility**		
Once a year	60	26.4
Twice a year	8	3.5
Frequently	36	15.9
Any time needed	68	30
Never	55	24.2
**Time taken to reach the nearest health facility**		
<40 min	106	46.7
40-79 min	77	33.9
80 min	44	19.4
**Health talks in the facility are very educative on FP**		
Yes	208	91.6
No	19	8.4
**Place of accessing FP services**		
Nearby health center	144	63.4
At a national referral hospital	25	11
Do not use FP at all	44	19.4
Others	14	6.2

### Practices on family planning of women attending antenatal clinic at KNH

Table 2 shows that 29.9% of the pregnancies were unintended out of which 83.8% were mistimed and 16.2% were unwanted pregnancy. 70% of the pregnancies the women had were intended. Contraceptive use before the current pregnancy was reported among 42.3% of the women. The most popular method among them was pills (40.6%) followed by injections 26%. Long term reversible contraceptives (LARC) like IUD and Implants were used by 16.7% and 11.5% of the respondents, respectively. A substantial proportion (41%) said they did not use contraceptives even when they did not intend to get pregnant while 17.2% practiced traditional contraceptive methods. In terms of decision-making for pregnancy, 77.1% said they both (respondent and the sexual partner) made the decision while 20.7% said it was dependent on the woman. Regarding contraceptives utilization, the decision was made by 48.5% of the women and 41.0% of the study participants involved their sexual partners in decision making.

**Table 2.** Practices on family planning practices among study participants.

Categorical variable	Frequency (n=227)	Percentage (%)
**Pregnancy intentions**		
Wanted to become pregnant then	159	70
Wanted to become pregnant later	57	25.1
Did not want any more children	11	4.8
**Unintended pregnancy**		
Yes	68	30
No	159	70
**Use of contraceptive methods**		
Yes	96	42.3
No	131	57.7
**Contraceptive methods (n=96)**		
Pills	39	40.6
IUD	16	16.7
Injection	25	26
Implants	11	11.5
Male condoms	8	8.3
Female condoms	2	2.1
Rhythm/Natural method	9	9.4
Withdrawal	3	3.1
**Uses contraceptive when not intending to get pregnant**		
Yes	134	59
No	93	41
**Practices traditional family methods**		
Yes	39	17.2
No	188	82.8
**Decision-maker on when to get pregnant**		
Husband/Sexual partner	5	2.2
Herself	47	20.7
Both	175	77.1
**Decision-maker on contraceptive use**		
Husband/Sexual partner	10	4.4
Herself	110	48.5
Both	93	41
None	14	6.2

### Facility-based factors among study participants

Table 3 shows that out of all the study participants, 73.6% of them sought the family planning services from health facilities. 24.2% of the women had never accessed Family Planning services. For those who sought the services, 26.4% accessed them once a year while 30% looked for the services whenever they needed. Most (46.7%) of the women lived in closed proximity to a health care facility. Health talks were useful in informing on family planning according to 91.6% of the women and 63.4% said they accessed the services from the nearest health facility.

**Table 3.** Facility-based factors among the study participants.

Variable	n (%)
**Frequency of seeking FP services in health facility**	
Once a year	60 (26.4)
Twice a year	8 (3.5)
Frequently	36 (15.9)
Any time needed	68 (30.0)
Never	55 (24.2)
**Time taken to reach the nearest health facility**	
<40 min	106 (46.7)
40-79 min	77 (33.9)
80 min	44 (19.4)
**Health talks in the facility are very educative on FP**	
Yes	208 (91.6)
No	19 (8.4)
**Place of accessing FP services**	
Nearby health center	144 (63.4)
At a national referral hospital	25 (11.0)
Do not use FP at all	44 (19.4)
Others	14 (6.2)

### Factors associated with unintended pregnancy among pregnant women attending antenatal care clinic at KNH

As shown in Table 4, there was an incresed risk of unintended pregnancies among women aged below 25 years compared to the 35+ years, OR 5.2 (95% CI 2.0-13.2), p<0.001. Those who were not married were twice more likely to report unintended pregnancies, OR 2.9 (95% CI 1.4-5.8), p=0.003. Other demographic factors such as rural or urban residence, woman’s or partner’s level of education, occupation and religion were not associated with pregnancy intentions.

**Table 4.** Bivariate analysis: Association between pregnancy intention and the background characteristics among women attending antenatal clinic at KNH.

Variable	Pregnancy intention		OR (95% CI)	p value<0.05
	Unintended (n=68)	Intended (n=159)		
**Age in years**				
<25	20 (29.4)	13 (8.2)	5.2 (2.0-13.2)	**<0.001**
25-29	16 (23.5)	41 (25.8)	1.3 (0.6-3.1)	0.519
30-34	19 (27.9)	61 (38.4)	1.1 (0.5-2.4)	0.898
35+	13 (19.1)	44 (27.7)	1.0	
**Residence**				
Urban	60 (88.2)	137 (86.2)	1.2 (0.5-2.9)	0.673
Rural	8 (11.8)	22 (13.8)	1.0	
**Education level**				
Primary	3 (4.4)	14 (8.8)	0.4 (0.1-1.5)	0.161
Secondary	19 (27.9)	58 (36.5)	0.6 (0.3-1.2)	0.134
Post-secondary	46 (67.6)	87 (54.7)	1.0	
**Marital status**				
Not married	19 (27.9)	19 (11.9)	2.9 (1.4-5.8)	**0.003**
Married	49 (72.1)	140 (88.1)	1.0	
**Religion**				
Catholic	30 (44.1)	74 (46.5)	1.0	
Protestant	28 (41.2)	79 (49.7)	0.9 (0.5-1.6)	0.663
Muslim	5 (7.4)	3 (1.9)	4.1 (0.9-18.3)	0.105
Others	5 (7.4)	3 (1.9)	4.1 (0.9-18.3)	0.105
**Partner’s education level**				
None	2 (2.9)	1 (0.6)	5.2 (0.5-58.4)	0.199
Primary	5 (7.4)	3 (1.9)	4.3 (1.0-18.8)	0.052
Secondary	16 (23.5)	46 (28.9)	0.9 (0.5-1.8)	0.749
Post-secondary	40 (58.8)	103 (64.8)	1.0	
Do not know	5 (7.4)	6 (3.8)	2.1 (0.6-7.4)	0.219
**Occupation**				
Employed	41 (60.3)	104 (65.4)	1.0	
Unemployed	27 (39.7)	55 (34.6)	1.2 (0.7-2.2)	0.462
**Gravidity**				
Once	21 (30.9)	23 (14.5)	2.8 (1.4-5.9)	**0.004**
2 times	19 (27.9)	49 (30.8)	1.2 (0.6-2.4)	0.591
3 and more	28 (41.2)	87 (54.7)	1.0	
**Parity**				
Zero	16 (23.5)	22 (13.8)	1.5 (0.6-3.4)	0.391
Once	17 (25.0)	46 (28.9)	0.7 (0.3-1.6)	0.455
2 times	17 (25.0)	55 (34.6)	0.6 (0.3-1.4)	0.228
3 and more	18 (26.5)	36 (22.6)	1.0	
**Use of contraceptive methods**				
Yes	41 (60.3)	55 (34.6)	2.9 (1.6-5.2)	**<0.001**
No	27 (39.7)	104 (65.4)	1.0	
**Decision-maker on pregnancy**				
Husband/Sexual partner	3 (4.4)	2 (1.3)	5.4 (0.8-33.5)	0.079
Herself	27 (39.7)	20 (12.6)	4.9 (2.5-9.6)	**<0.001**
Both	38 (55.9)	137 (86.2)	1.0	
**Decision-maker on FP use**				
Husband/Sexual partner	5 (7.4)	5 (3.1)	5.6 (1.4-22.1)	0.006
Herself	46 (67.6)	64 (40.3)	4.1 (2.0-8.0)	**<0.001**
Both	14 (20.6)	79 (49.7)	1.0	
None	3 (4.4)	11 (6.9)	1.5 (0.4-6.2)	0.543
**Frequency of seeking FP services**				
Once a year	9 (13.2)	51 (32.1)	1.0	
Twice a year	2 (2.9)	6 (3.8)	1.9 (0.3-1.9)	0.471
Frequently	8 (11.8)	28 (17.6)	1.6 (0.6-4.7)	0.369
Any time needed	25 (36.8)	43 (27.0)	3.3 (1.4-7.8)	0.005
Never	24 (35.3)	31 (19.5)	4.4 (1.8-10.6)	**0.001**
**Time taken to reach the nearest health facility**				
<40 min	27 (39.7)	79 (49.7)	1.0	
40-79 min	28 (41.2)	49 (30.8)	1.7 (0.9-3.2)	0.113
80 min	13 (19.1)	31 (19.5)	1.2 (0.6-2.7)	0.607
**Health talks in the facility are very educative on FP**				
Yes	57 (83.8)	151 (95.0)	1.0	
No	11 (16.2)	8 (5.0)	3.6 (1.4-9.5)	**0.005**
**Place of accessing FP services**				
Nearby health center	40 (58.8)	104 (65.4)	1.0	
At a national referral hospital	9 (13.2)	16 (10.1)	1.5 (0.6-3.8)	0.403
Do not use FP at all	16 (23.5)	28 (17.6)	1.5 (0.7-3.0)	0.276
Others	3 (4.4)	11 (6.9)	0.7 (0.2-2.7)	0.610

Gravidity was significantly associated with pregnancy intentions with gravida 1 more likely to have unintended pregnancies [OR 2.8 (95% CI 1.4-5.9), p=0.004] compared to those with gravida 3 or more (OR1.0). However, parity did not show any significant association with pregnancy intentions.

In addition, the women who used contraceptive methods prior to the current pregnancy were more likely to report unintended pregnancies, OR 2.9 (95% CI 1.6-5.2), p<0.001. In relation to decision making for pregnancy and Family Planning use, there was a higher likelihood of unintended pregnancies in relationships where the woman was the only decision maker on when to get pregnant [OR 4.9 (95% CI 2.5-9.6), p<0.001] and FP use [OR 4.1 (95% CI 2.0-8.0), p<0.001] compared to those with both partners involved.

Unintended pregnancies was likely to be reported among women who never sought FP services from health facilities [OR 4.4 (95% CI 1.8-10.6), p=0.001] and those who sought the services only when they felt they needed it [OR 3.3 (95% CI 1.4-7.8), p=0.005]. Also, the women with unintended pregnancies thought health talks were not educative on FP, OR 3.6 (95% CI 1.4-9.5), p=0.005.

### Predictors of unintended pregnancies

Table 5, shows a multivariate logistic regression revealing that ages less than 25 years [aOR 8.1 (95% CI 1.4-48.6), p=0.022], prior use of family planning methods [aOR 7.9 (95% CI 2.5-25.0), p<0.001, the woman independently deciding on when to get pregnant [aOR 3.8 (95% CI 1.3-11.2), p=0.014], never seeking FP service from a health facility [aOR 4.4 (95% CI 1.0-19.6), p=0.050] and the attitude that health talks are not useful in informing on FP [aOR 5.6 (95% CI 1.1-27.6), p=0.033] were suggestively associated with unintended pregnancy. Marital status and gravidity of the women were not independently related with occurrence of unintended pregnancies.

**Table 5.** Multivariate logistic regression analysis: Factors associated with unintended pregnancy among women attending Antenatal clinic at Kenyatta National Hospital, Nairobi.

Variable	Adjusted OR (95% CI)	p value<0.05
**Age in years**		
<25	8.1 (1.4-48.6)	**0.022**
25-29	1.6 (0.4-5.7)	0.498
30-34	1.1 (0.3-3.4)	0.910
35+	1.0	
**Use of contraceptive methods**		
Yes	7.9 (2.5-25.0)	**<0.001**
No	1.0	
**Decision-maker on pregnancy**		
Husband/Sexual partner	4.3 (0.2-98.4)	0.359
Herself	3.8 (1.3-11.2)	**0.014**
Both	1.0	
**Frequency of seeking FP services**		
Once a year	1.0	
Twice a year	1.6 (0.1-27.7)	0.738
Frequently	1.0 (0.2-4.7)	0.989
Any time needed	2.0 (0.5-7.1)	0.299
Never	4.4 (1.0-19.6)	**0.050**
**Health talks in the facility are very educative on FP**		
Yes	1.0	
No	5.6 (1.1-27.6)	**0.033**

## Discussion

This study sought to find out the proportion and determinants of unintended pregnancy in among women attending antenatal clinic. Our finding revealed that 29.9% of these women had unintended pregnancy. This proportion of women was slightly lower compared to the proportion of unintended pregnancy in Kenya from reports from other studies like in 2012 was at 40% ( Mumah *et al.*, 2013), 34.4% ( Ameyaw *et al.*, 2019) and 41.9% ( Monitoring, 2020). This means that about 29.9% of women attending antenatal clinic are at risk of having unintended pregnancy and further more exposed to the severe health implications associated with it.

In this study, younger women were at a higher risk of unintended pregnancies with those aged below 25 years were 8 times likely to report unintended pregnancy compared to those aged 35 years and above. Similar findings were reported in a previous study in Nairobi that showed younger women having a greater possibility of getting unintended pregnancies ( Ikamari *et al.*, 2013). This may be because the younger women were more exposed to frequent sexual intercourse with lower utilization or higher failure rates of contraceptives ( Habib *et al.*, 2017).

Marital status did not have association with unintended pregnancies in this research despite those who were not married showing a higher risk. This significant finding was lost when the results were adjusted for other characteristics including age. This was explained by the general trend that majority of the unmarried women were younger in age which came out as an important factor associated with unintended pregnancies. Studies in Malaysia and South Africa have reported that unintended pregnancies were higher in women who were not married (Yusof *et al.*, 2018; Haffejee *et al.*, 2018).

Studies elsewhere and in a population in Kenya have shown increased risk of unintended pregnancies among women with higher parity. Unintended pregnancies were more likely in women with para 4 and above in Tanzania ( Exavery *et al.*, 2014). A study in Kenya showed 2.5 fold risk of unintended pregnancies among women with parity of three and above ( Ikamari *et al.*, 2013). However, this study did not find any association between parity and occurrence of unintended pregnancies.

The association between lower gravidity and the pregnancies being reported as unintended but was lost when adjusted for other significant characteristics. Younger women had lower numbers of previous pregnancies hence age was independently associated with unintended pregnancy thus eliminating gravidity. According to this study, other demographic factors such as rural or urban residence, partner’s level of education, occupation and religion were not significantly associated with pregnancy intentions.

Decisions to use of contraceptive methods was determined by the pregnancy intentions of the woman and partly the sexual partner. About two-fifths of the women in this study reported use of contraceptive methods in the period before the current pregnancy. This was comparable to the proportion reported in a survey in Kenya where 42.6% of the women used at least a method of contraceptive to avoid pregnancy (KDHS 2014). Only 59% of the women in this study said they use contraceptives to avoid pregnancies. Pills and injectables were the most utilized methods in this study and this is quite similar to the Kenya national demographic survey report that showed that injectables was the most preferred contraceptive methods with a quarter of the women using the method (KDHS 2014). In 2018, a survey also indicated that 47.2% of women aged 15-49 years use injectables and pills as their preferred contraceptive methods ( Monitoring, 2020).

In addition, this study found that the decision to use contraceptives was dependent mainly on the woman while conception intentions were discussed in most circumstances between the two partners. The relationships in which the woman was the sole decision-maker regarding conception had a 4-fold risk of unintended pregnancies. This is comparable to the findings of a study that was carried out in Bangladesh where women who discussed their intent of using contraceptives with their sexual partners showed less prevalence of unintended pregnancy likened to those who did not discuss at all (28.4% vs 31.1%) (Kamal & Islam, 2011). This could be attributed to the lack of a common goal within the relationship hence conflicting intentions. In certain societies the social norm limits women freedom to make choices and are fully dependent on their male sexual partners or spouses. So in certain relationships men make decision on health seeking behavior of the sexual partner or spouse including use of contraceptives ( Ali, Ali, *et al.*, 2016).

Our study showed that utilization of contraceptives before pregnancy predicted occurrence of unintended pregnancies. Women who used contraceptives had an eight-fold risk of unplanned pregnancies. At the same time, women having unintended pregnancies were 4 times more likely to have never sought family planning services in a health care facility. This means the women utilizing contraceptive methods were not receiving adequate efficacy because lack of expert advice on the uptake and incorrect use of the methods. Other researchers have found that unintended pregnancies were related to lack of use of contraceptives as well as incorrect use or failure of the methods (Kassie *et al.*, 2017).

In Kenya, family planning programs greatly impact on unintended pregnancy because the quality of care is determined by accessibility and availability of contraceptives. 80% of the pregnant women agreed that the nurses and doctors explained to them reproductive health issues and handled them with a lot of confidentiality. 63% of the women who experienced unintended pregnancy thought that they experienced that because of family planning failure (7%), mistake (34.9%) and 34.9% again could not explain why. This could also be supported by the fact that those who reported unintended pregnancies thought the health talks in the clinics were not meeting their need regarding family planning. This attitude could impact on their healthcare-seeking behavior. This study found out that women who never sought family planning services had a four-fold increased chance of unintended pregnancies but distance to Family Planning facility had no significant association. Kenya family planning programs in collaboration with Ministry of Health and other Reproductive health stakeholders need to continue with the Family planning 2020 campaigns on the scaling up of long-term reversible methods of family planning.

## Conclusions

In conclusion, despite the high level of education, majority living in town and the fact that most of these women attend antenatal clinic when pregnant, the proportion of unintended pregnancy is significantly high. Age below 25 years, the woman being the only decision maker on when to conceive, never seeking Family Planning (FP) service from a health facility, attitude that health talks are not useful in informing Family Planning and women who get pregnant while using contraceptives are likely to report the pregnancy as unintended. There is need towards integrating Sustainable family planning health education and promotion with antenatal care services to increase awareness of consequences of unintended pregnancy, factors associated with it and how to prevent it through modern family planning.

### Limitations of this study

The small sample size may affect the application of these findings to the whole general population. There may have been some response bias influenced by social desirability bias that inclined the women to report their pregnancy as intended even though it was not, leading to an under- estimation of the burden of unintended pregnancies.

## Data availability

Figshare. Determinants of unintended pregnancy among women attending antenatal clinic at Kenyatta National Hospital. DOI: https://doi.org/10.6084/m9.figshare.19403879.v1 ( Ojuok *et al.*, 2022).

This project contains the following underlying data:

- A cross-sectional study design was used. Data was collected using a structured and interviewer guided questionnaire from 227 participants. The prevalence and determinants of unintended pregnancy was calculated with bivariate and multivariate logistic regressions.
- This data sought to find the prevalence of unintended pregnancy among the study population and the determinants of unintended pregnancy.

Data are available under the terms of the Creative Commons Attribution 4.0 International license (CC-BY 4.0).

## References

[ref1] Ali SA Ali SA : Unmet need for contraception and unintended pregnancies among women of reproductive age group: A situation analysis. *El Mednifico Journal.* 2014;2(3):259. 10.18035/emj.v2i3.242

[ref2] Ali SA Ali SA Khuwaja NS : Determinants of Unintended Pregnancy among Women of Reproductive Age in Developing Countries: A Narrative Review. *Journal of Midwifery and Reproductive Health.* 2016;4(1):513–521. 10.22038/jmrh.2016.6206

[ref3] Ali SA Tikmani SS Qidwai W : *Prevalence and determinants of Unintended Pregnancy: Systematic Review. July.* 2016.

[ref4] Ameyaw EK Budu E Sambah F : Prevalence and determinants of unintended pregnancy in sub-Saharan Africa: A multi-country analysis of demographic and health surveys. *PLoS One.* 2019;14(8):e0220970. 10.1371/journal.pone.0220970 31398240 PMC6688809

[ref5] Daniel WW Cross CL : *Biostatistics: A foundation for Analysis in the Health Sciences.* 2013. 10.1017/CBO9781107415324.004

[ref6] Exavery A Kanté AM Njozi M : Predictors of mistimed, and unwanted pregnancies among women of childbearing age in Rufiji, Kilombero, and Ulanga districts of Tanzania. *Reproductive Health.* 2014;11(1):1–9. 10.1186/1742-4755-11-63 25102924 PMC4130430

[ref7] Grenier L Suhowatsky S Kabue MM : Impact of group antenatal care (G-ANC) versus individual antenatal care (ANC) on quality of care, ANC attendance and facility-based delivery: A pragmatic cluster-randomized controlled trial in Kenya and Nigeria. *PLoS One.* 2019;14(10):e0222177–e0222118. 10.1371/journal.pone.0222177 31577797 PMC6774470

[ref8] Habib MA Raynes-Greenow C Nausheen S : Prevalence and determinants of unintended pregnancies amongst women attending antenatal clinics in Pakistan. *BMC Pregnancy Childbirth.* 2017;17(January):156. 10.1186/s12884-017-1339-z 28558671 PMC5450067

[ref9] Hamdela B Mariam AG Tilahun T : Unwanted pregnancy and associated factors among pregnant married women in Hosanna town, Southern Ethiopia. *PLoS One.* 2012;7(6) 10.1371/journal.pone.0039074

[ref10] Hussain R : Abortion and unintended pregnancy in Kenya. *Issues Brief (Alan Guttmacher Inst.).* 2012;2008(2):1–4. 10.1067/j.cpsurg.2012.08.001 22734165

[ref11] Ikamari L Izugbaru C Oghako R : Prevalence and determinants of unintended pregnancies in Nairobi, Kenya. *BMC Pregnancy Childbirth.* 2013;13(1):2014. 10.1186/1471-2393-13-69 23510090 PMC3607892

[ref12] Literature Review, Conceptual Framework and Methodology Background Characteristics of Women:n.d.

[ref13] Monitoring, P: *Pma2020/kenya. 2018.* 2020. (December 2018), 2018–2019.

[ref14] Mumah J Kabiru C Mukiira C : *Unintended Pregnancies in Kenya: A Country Profile. April.* 2013;110. Reference Source

[ref15] Nyarko SH : Unintended Pregnancy among Pregnant Women in Ghana: Prevalence and Predictors. *J. Pregnancy.* 2019;2019:1–8. 10.1155/2019/2920491 PMC6374858 30834145

[ref16] Obiero BO Mwai D : Determinants of Unintended Pregnancy in Kenya. *International Journal of Academic Research in Economics and Management Sciences.* 2018;7(1). 10.6007/IJAREMS/v7-i1/3875

[ref20] Ojuok R Mutai J Dr Nyamongo D Dr : Determinants of unintended pregnancy among women attending antenatal clinic at Kenyatta National Hospital. figshare. *Dataset.* 2022. 10.6084/m9.figshare.19403879.v1 PMC9606630 36329794

[ref17] Sedgh G Bankole A Oye-Adeniran B : Unwanted pregnancy and associated factors among Nigerian women. *Int. Fam. Plan. Perspect.* 2006;32(4):175–184. 10.1363/ifpp.32.175.06 17237014

[ref18] UNFPA: *Kenya Annual Report 2015.* 2015.

[ref19] UNFPA: *Summary report of the assessment of UNFPA’s advocacy campaign to end preventable maternal and Newborn mortality in Kenya.* 2016;1–20.

