# Supplementary material for: Determinants of unintended pregnancy among women attending antenatal clinic at Kenyatta National Hospital
Source: F1000Res. 2022 May 27;11:585. [Version 1] doi: 10.12688/f1000research.108815.1 (PMC9606630; doi:10.12688/f1000research.108815.1)
Supplement: Peer review history

# Peer review: Reviewer response for version 1

**DOI:** 10.5256/f1000research.120244.r153526

**Published:** 2022-10-26

**Authors:** Chitkasaem Suwanrath

**Affiliations:** 1 Prince of Songkla University, Hat Yai, Thailand

This research is interesting and useful for developing country where unintended pregnancy is prevalent.

- The title is appropriate and reflects the study design as well as prediction of potential factors associated with the unintended pregnancy.
- The abstract is well written. The background states the magnitude of the problem and gap of knowledge. Methods, results and conclusions are appropriate and concise. However, I would suggest to delete the word "such as" in line 3 of Results. Since there are only 3 significant factors.
- keywords: in my opinion, "18-49 years" should be deleted.
- Introduction: It is well written, reflecting the magnitude of the problem in regions with high prevalence of unintended pregnancy as well as the authors' country. Gap of knowledge is stated. Literature review is appropriate and relevant to the study.
- Methods: Study design is appropriate. Sample size calculation is correct. Sampling technique is clearly described. Data analysis is well explained.
- Results: All relevant outcomes are presented. However, please do not replicate the results in the text if they are already in the tables, just summarize which factors are statistically significant.
- Discussion are appropriate, summarized significant findings and compared with relevant previous studies. It is well explained for associated factors with unintended pregnancy.
- Conclusions are concise with good recommendation.

Is the work clearly and accurately presented and does it cite the current literature?

Yes

If applicable, is the statistical analysis and its interpretation appropriate?

Yes

Are all the source data underlying the results available to ensure full reproducibility?

Yes

Is the study design appropriate and is the work technically sound?

Yes

Are the conclusions drawn adequately supported by the results?

Yes

Are sufficient details of methods and analysis provided to allow replication by others?

Yes

Reviewer Expertise:

Maternal fetal medicine. Obstetrics and Gynecology

I confirm that I have read this submission and believe that I have an appropriate level of expertise to confirm that it is of an acceptable scientific standard.

# Peer review: Reviewer response for version 1

**DOI:** 10.5256/f1000research.120244.r148558

**Published:** 2022-09-13

**Authors:** Ayodele. O. Arowojolu

**Affiliations:** 1 University of Ibadan, Ibadan, Nigeria

- The title is appropriate and relevant to the study carried out. It predicts the content and type of study.  
- The abstract is structured, reflecting the introduction, methodology, results, and conclusion of the study.  The keywords are relevant.
- The introduction is clearly and accurately presented and does cite the current literature.  
- The study design is appropriate, and the work has academic merit.  
- The details of the methods show that the study is a survey using a structured questionnaire (of unknown source). However, it is uncertain if this was self-administered or not.  (The authors dwelt more on the process of obtaining consent from the participants by the assisting nurses).  
- Sample size calculation used appropriate formula, but no correction was made for attrition during the study.  Fortunately, no attrition occurred during the study.
- The sampling technique is systematic using recorded antenatal case record as the sample frame with calculated sampling interval.
- The statistical analysis and its interpretation are appropriate. The section is well described and should allow replication by others. However, the analysis will be richer if a stepwise multivariate logistic regression is performed in addition, to determine the influence of the relevant variables in predicting the outcome measure (unintended pregnancy). All the source data underlying the results are available to ensure full reproducibility of data.   
- The results are clearly presented in the text and tables with correctly labeled titles.  Statistical measures and results are well shown.
- The discussion of all the findings of the study according to the stated objectives is adequate and related to previous studies in the literature with which appropriate comparisons are made.  The validity of the result findings is discussed along with adequate explanation of the factors associated with them.  
- The conclusions drawn are adequately supported by the results provided. The recommendation of family planning health education and improvement of facilities, as well as promotion of antenatal care services usage in the community, so as to increase awareness of consequences of unintended pregnancy and its prevention is appropriate.

Is the work clearly and accurately presented and does it cite the current literature?

Yes

If applicable, is the statistical analysis and its interpretation appropriate?

Yes

Are all the source data underlying the results available to ensure full reproducibility?

Yes

Is the study design appropriate and is the work technically sound?

Yes

Are the conclusions drawn adequately supported by the results?

Yes

Are sufficient details of methods and analysis provided to allow replication by others?

Yes

Reviewer Expertise:

Public health Nutrition and clinical nutrition, Contraception and reproductive health issues.

I confirm that I have read this submission and believe that I have an appropriate level of expertise to confirm that it is of an acceptable scientific standard.
